# Supplementary material for: MAP4K3 inhibits Sirtuin-1 to repress the LKB1–AMPK pathway to promote amino acid-dependent activation of the mTORC1 complex
Source: Life Sci Alliance. 2023 May 23;6(8):e202201525. doi: 10.26508/lsa.202201525 (PMC10205607; doi:10.26508/lsa.202201525)
Supplement: Supplementary file 3 [file LSA-2022-01525_TableS1.docx]

**Supplementary Table 1. Sirtuin-1 phosphorylation sites detected by mass spectrometry**

|  |
| --- |
| S26 |
| S27 |
| S47 |
| **T344** |
| **T530** |
| S535 |
| S538 |
| S539 |
| S540 |
| S545 |
| S562 |
| S569 |
| S590 |
| S605 |
| S615 |
| **T719** |
| S747 |
